# Development of allogeneic iPS cell‐based therapy: from bench to bedside

**DOI:** 10.15252/emmm.202115315

**Published:** 2022-12-07

**Authors:** David H McKenna, Rita C R Perlingeiro

**Affiliations:** ^1^ Molecular and Cellular Therapeutics University of Minnesota Saint Paul MN USA; ^2^ Stem Cell Institute University of Minnesota Minneapolis MN USA; ^3^ Department of Medicine, Lillehei Heart Institute University of Minnesota Minneapolis MN USA

**Keywords:** Stem Cells & Regenerative Medicine

## Abstract

This commentary provides a brief overview of the steps necessary for the generation of an induced pluripotent stem (iPS) cell‐derived clinical grade product. This process requires extensive, proper documentation as well as a thoughtful and systematic optimization of the manufacturing methods to ensure maintenance of the key biological features of the product, compliance with current good manufacturing practices (cGMP), and most importantly patient safety. The scale‐up and optimization also ideally include the identification of efficient and cost‐effective purification/isolation and expansion of the target cell population.

## Graphical abstract

This Commentary discusses the production process of an induced pluripotent stem cell‐derived clinical grade therapeutic – from bench to bedside.

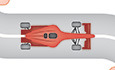

The inception of human‐induced pluripotent stem (iPS) cells from somatic tissue by Yamanaka and colleagues (Takahashi *et al*, 2007) in 2007 triggered a new wave of enthusiasm and hope for the field of regenerative medicine. Now, 15 years later, these feelings are stronger than ever as several iPS cell‐based therapies have advanced from the bench to the bedside. Several clinical trials are underway in the USA and Japan involving both allogeneic and autologous transplantation of iPS cell derivatives. These include iPS cell‐derived retinal pigment epithelium cell sheets for the treatment of macular degeneration (Araki *et al*, 2019; Jha *et al*, 2021; ClinicalTrials.gov Identifier: NCT04339764, NCT02464956, UMIN000011929), iPS cell‐derived dopaminergic neurons for the treatment for Parkinson's disease (UMIN000033564), and iPS cell‐derived NK cells as an immunotherapy regimen for the treatment of solid tumors and hematological disorders (Hermanson *et al*, 2016; Cichocki *et al*, 2020; NCT03841110, NCT04245722, NCT04023071). To date, results indicate safety, supporting the use of iPS cell derivatives for the treatment of several diseases (Yamanaka, 2020).

Despite evident translational progress, the pipeline of completed/ongoing clinical trials using iPS cells is still anemic considering the robust literature documenting preclinical proof‐of‐concept for the regenerative potential of iPS cell derivatives using numerous mouse models of disease. Many reasons could account for this conundrum, but foremost among these may be the complex road to translation, which is foreign territory to most basic scientists. Here, we provide an overview of the main steps.

## Where to start?

**Figure 1:**
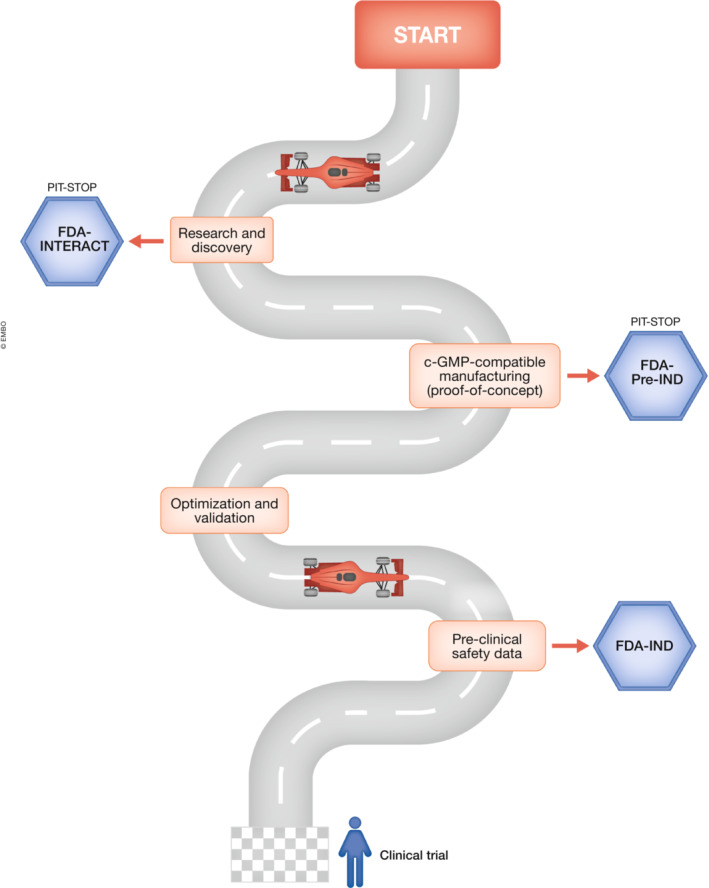
Translational development of cellular therapies Schematic overview depicting the overall steps of clinical development. Safety data may be collected earlier in the process (e.g., prior to optimization and validation) or in parallel to another step(s).

A good starting point is to identify where the research is on the roadmap for translation (Fig 1). How robust is the evidence for therapeutic potential? Are there processes that need to be refined, such as the *in vitro* differentiation protocol and/or purification strategy? These are critical points to address prior to embarking on preclinical development. Most investigators will find themselves at the research/discovery stage, that is, at the very beginning of the road, even in cases when the technology has been extensively validated through *in vivo* preclinical scientific studies, that have been peer‐reviewed and published.

## Decisions to make prior to preclinical development

Both allogeneic and autologous cell therapies have particular advantages for clinical development and application. The autologous product is attractive as it allows for personalized medicine and obviates concern for rejection. The allogeneic approach is advantageous as it allows for an “off the shelf” delivery and thus can be tested in multiple patients. In addition, the allogeneic or third‐party product is typically more cost‐effective and lends itself to a more standardized treatment and manufacturing process under cGMP. Expertise gained from the production of allogeneic cells can be applied to the manufacturing of subsequent autologous cell products.

If opting for an allogeneic/third‐party cell therapy product, the selection of the iPS cell line to be used is a critical decision. Ideally, iPS cells that are chosen should be available as both research and cGMP grade. The research grade cells will support preclinical studies prior to the switch to the cGMP version for clinical manufacturing in support of the phase 1 trial. Of course, prior to any movement toward cGMP manufacturing, the investigators should first assess the feasibility of the research grade iPS cell line to efficiently differentiate into the specific cell type of interest. Additionally, in order to minimize costs, it is advisable to perform optimization studies in the research laboratory, with guidance from experts in chemistry, manufacturing, and controls (CMC), before starting production at the cGMP laboratory with the cGMP grade iPS cell line. The cost of the iPS cell line acquisition and freedom to operate are also critical considerations for budgeting and development. Rather sooner than later, it is important to verify that all required documents for the candidate iPS cell line are in place for clinical application, including donor informed consent, IRB approval, and donor screening. The latter comprises of medical history and examination, as well as blood testing, which serves to minimize the risk of potential transmission of adventitious agents (more on this aspect below in certification of analysis).

Prior to execution of cGMP manufacturing and testing, it is advisable to consult the FDA though a pre‐investigational new drug (pre‐IND) application to receive feedback and guidance on product development, testing, and clinical trial design plans. If the investigational product has been identified and some proof‐of‐concept studies have been completed, but definitive toxicology studies have not yet been designed or conducted, the investigators may benefit from an INTERACT (INitial Targeted Engagement for Regulatory Advice on CBER ProducTs) meeting with FDA at this early stage (Fig 1). This type of meeting replaced the so‐called “pre‐pre‐IND” meeting.

## Manufacturing of an iPS cell‐derivative for clinical translation

To translate basic research to clinical application, culture methodologies must be adapted into a cGMP/clinically appropriate protocol. An initial step in this process is to identify and perform a risk assessment of all reagents and supplies in the manufacturing process. cGMP or USP‐grade materials should always be used when available. Research grade materials may be used with agreement from the FDA; however, specific testing and/or additional testing may be required by the Agency. It is ideal to eliminate or replace any component of animal origin with a chemically defined cGMP substitute reagent. However, if it can be demonstrated that a culture component of animal origin is essential, it is usually permitted. For example, when fetal bovine serum (FBS) is deemed necessary for manufacturing, FDA requires it be sourced from a qualified herd from a country considered free of risk of transmissible spongiform encephalopathy (TSE).

Another important aspect is purification of the target cell population, not only for efficiency but also for safety to avoid the presence of potential contaminating residual undifferentiated pluripotent stem cells in the final product, which in theory could give rise to teratomas. As we discuss below, this risk can be mitigated though rigorous release criteria and properly designed safety studies that address tumorigenicity. For purification, the use of one single surface marker that identifies the target cell population is preferred over multiple markers due to cost and clinical compatibility as this can usually be adapted to magnetic bead, which unlike fluorescence‐activated single cell sorting (FACS), allows for the use of a closed system.

Based on the institutional vision and the downstream plan for the clinical trial(s), the investigators or program leadership should try to anticipate the required number of vials and cells/vial for the master cell bank (MCB) and working cell bank (WCB). This is key to determine scalability and feasibility. For instance, it is important to determine how many culture vessels will be required and what type of vessel should be used? Expansion systems will likely need to be adapted to multilayer cell culture flasks/cell factories or bioreactors.

Another strategic decision involves the determination of activity of fresh vs. cryopreserved final product. The latter option makes the approach much more feasible for multicenter participation among distant sites and ultimately exportability. Preclinical studies should include the final product in the anticipated state, that is, fresh or frozen, to allow for finalization of the manufacturing plan for the trial.

Once the methodology is sufficiently developed into a clinically relevant protocol in the research laboratory, the technology should be transferred to the cGMP cell manufacturing facility for scale‐up, optimization, and ultimately performance qualification (PQ) or validation. The cGMP laboratory will start with engineering runs prior to locking down methods and executing the PQ. Standard operating procedures (SOPs) and a batch production record (BPR), as established by the PQ, are then used for clinical production. It is important to test and validate the cell product *in vitro* and *in vivo* at every step of the optimization process to ensure the clinical product remains equivalent to the product studied in the research laboratory.

## Certification of MCB and final cell product

A banking strategy is the standard approach for the manufacture of an iPS cell‐derived cell therapy product. First, a small (i.e., 10–20 vials) parent or source bank is established from starting material (e.g., neonatal foreskin fibroblasts, umbilical cord blood) or from one to two vials of an established cGMP iPS cell MCB. It is important to complete the donor eligibility determination as per the regulations (21CFR 1271) if you are establishing your own iPS cell bank. If not establishing an in‐house iPS cell line, it is advisable to confirm with the manufacturing group that the donor was deemed eligible as per FDA. This regulatory requirement of donor eligibility includes a health history questionnaire and standard testing for infectious disease markers using a donor sample (e.g., maternal blood sample if using neonatal foreskin fibroblasts or umbilical cord blood; U.S. Department of Health and Human Services Food and Drug Administration Center for Biologics Evaluation and Research, 2007). The quality control (QC) testing on the parent or source bank is typically limited and may include sterility, mycoplasma, and pluripotency characterization.

From the parent or source bank, a vial is thawed and placed in an expansion culture to establish the MCB. The MCB may be a standard iPS cell bank or a further modified bank (e.g., genetic manipulation), and the decision for the approach is a strategic one. The MCB is extensively characterized and includes testing for bacteria and fungal organisms (i.e., sterility), mycoplasma, and numerous viruses. In addition to testing for viruses of human origin, testing for viruses of nonhuman origin is included and based on the culture medium and reagents. For example, an MCB made using fetal calf serum will include assessment for the presence of bovine viruses. Master cell bank characterization testing is expensive and may easily cost greater than $100,000. Finally, an additional strategic decision is the determination of size of the MCB. The MCB must be able to support all preclinical and clinical work, which may include phase 1–3 trials and perhaps optimistically may need to support manufacture of a licensed product.

A WCB is the next tier of the banking strategy. A vial or a few vials from the well‐characterized MCB are thawed and used to initiate the manufacture of the WCB. The WCB is made with the same methods as the MCB; however, QC testing is limited by comparison. Working cell bank testing typically focuses on sterility and may be limited to sterility culture, endotoxin assay, and mycoplasma testing.

The last step with the banking strategy is the manufacture of the final cell therapy product. The details of the manufacturing schema will obviously be dependent on the clinical application. Likewise, much of the QC and/or lot release testing will be dependent on the targeted condition. Lot release testing may include the following: sterility culture, endotoxin assay, mycoplasma testing, viability, cytogenetics (G‐banding), and flow cytometry for markers informative of identity and possibly as part of a matrix approach for potency testing. Additional testing (which may be “for information only” in an early phase study) may include the following: whole‐genome sequencing, insertion site analysis, *in vitro* differentiation, *in vivo* regenerative potential, additional flow cytometry assessment for candidate markers of potency.

## IND‐enabling studies: biodistribution and safety

Prior to IND‐submission, investigators need to confirm that cell product is safe to be administered to patients. Major safety concerns include distribution to nontarget sites, inappropriate differentiation, inappropriate cell growth and teratoma development. Therefore, IND‐enabling studies are designed to address biodistribution/toxicology and tumorigenicity of a given cell product. Preclinical studies should recapitulate as close as possible the intended clinical scenario, including delivery site and cell dose. Immunodeficient mice are commonly used, and therefore recapitulating clinical dose may represent a challenge. If this is the case, investigators may need to provide a validated method of dose level extrapolation from mouse to human. Before initiating IND‐enabling studies, investigators should consult with regulatory agencies to make sure regulators are in agreement with proposed safety plans.

Ideally, these studies should be performed with the same batch of cells that are intended to be used in the future clinical trial. This is possible if the cell product is envisioned to be used off‐the‐shelf. If this is not the case, a cGMP cell product manufactured using the established SOPs and the same reagents (ideally same batches) can be used for IND‐enabling studies.

Moreover, it is recommended that biodistribution and safety studies to be conducted in accordance with good laboratory practices (GLP) to assure the validity, integrity, and reliability of preclinical safety data, which is a critical component of the IND package to be reviewed by regulatory agencies prior to clinical application approval. If the institution does not have a GLP‐certified facility, there are several preclinical contract research organizations (CROs) that provide this type of service.

## Conclusions

The future of iPS cell‐based regenerative medicine looks bright as more and more cell products make their way to the translational pipeline and eventually move into clinical trials. The translational road is winding, but thankfully, the path to cell therapy trials has already been blazed in the areas of hematopoietic and mesenchymal stem cell transplantation and cellular immune therapeutics such as CAR‐T cells. It has been our experience that engaging with colleagues with proficiency shepherding these types of cell therapy to the clinic, at a juncture as early as possible, can endow clinical translational iPS cell therapy programs with both the momentum and the proper direction necessary to bring them over the finish line. With proper guidance, the many aspects to be addressed become much less daunting. Therefore, if proof of concept has been clearly established for a given iPS cell‐derived product, investigators should seek guidance from experts in CMC and preclinical regulatory affairs. Some academic institutions have in‐house expertise with a clinical translational institute or translational cell therapy facility that can derive and expand cells under cGMP conditions, but most of the time, investigators may need to seek expertise and manufacturing facilities elsewhere. This should not be a reason for discouragement as there is a growing body of experts that provide consulting on translational projects and CROs that can execute the different tasks. As with any journey, the one for translation starts with a single step and is made shorter with good company.

## Disclosure and competing interests statement

DHM has no competing interests. RCRP is cofounder and holds equity in Myogenica.

## References

[emmm202115315-bib-0001] Araki H , Miura F , Watanabe A , Morinaga C , Kitaoka F , Kitano Y , Sakai N , Shibata Y , Terada M , Goto S *et al* (2019) Base‐resolution methylome of retinal pigment epithelial cells used in the first trial of human induced pluripotent stem cell‐based autologous transplantation. Stem Cell Reports 13: 761–774 31564644 10.1016/j.stemcr.2019.08.014 PMC6829753

[emmm202115315-bib-0002] Cichocki F , Bjordahl R , Gaidarova S , Mahmood S , Abujarour R , Wang H , Tuininga K , Felices M , Davis ZB , Bendzick L *et al* (2020) iPSC‐derived NK cells maintain high cytotoxicity and enhance in vivo tumor control in concert with T cells and anti‐PD‐1 therapy. Sci Transl Med 12: eaaz5618 33148626 10.1126/scitranslmed.aaz5618 PMC8861807

[emmm202115315-bib-0003] Hermanson DL , Bendzick L , Pribyl L , McCullar V , Vogel RI , Miller JS , Geller MA , Kaufman DS (2016) Induced pluripotent stem cell‐derived natural killer cells for treatment of ovarian cancer. Stem Cells 34: 93–101 26503833 10.1002/stem.2230 PMC4713309

[emmm202115315-bib-0004] Jha BS , Farnoodian M , Bharti K (2021) Regulatory considerations for developing a phase I investigational new drug application for autologous induced pluripotent stem cells‐based therapy product. Stem Cells Transl Med 10: 198–208 32946199 10.1002/sctm.20-0242 PMC7848308

[emmm202115315-bib-0005] Takahashi K , Tanabe K , Ohnuki M , Narita M , Ichisaka T , Tomoda K , Yamanaka S (2007) Induction of pluripotent stem cells from adult human fibroblasts by defined factors. Cell 131: 861–872 18035408 10.1016/j.cell.2007.11.019

[emmm202115315-bib-0006] U.S. Department of Health and Human Services Food and Drug Administration Center for Biologics Evaluation and Research (2007) Guidance for industry – eligibility determination for donors of human cells, tissues, and cellular and tissue‐based products (HCT/Ps)

[emmm202115315-bib-0007] Yamanaka S (2020) Pluripotent stem cell‐based cell therapy‐promise and challenges. Cell Stem Cell 27: 523–531 33007237 10.1016/j.stem.2020.09.014

